# Single-nucleus transcriptomics reveals the cellular immune responses to *Candidatus* Liberibacter asiaticus in rough lemon

**DOI:** 10.1093/hr/uhaf265

**Published:** 2025-10-01

**Authors:** Xu-Bin Tian, Jinhuan Zhou, Jiaxin Li, Yayu Li, Changyong Zhou, Zhen Song

**Affiliations:** National Citrus Engineering Research Center, Citrus Research Institute, Southwest University, Beibei, Chongqing 400715, China; Integrative Science Center of Germplasm Creation in Western China, Southwest University, Beibei, Chongqing 400715, China; National Citrus Engineering Research Center, Citrus Research Institute, Southwest University, Beibei, Chongqing 400715, China; Integrative Science Center of Germplasm Creation in Western China, Southwest University, Beibei, Chongqing 400715, China; National Citrus Engineering Research Center, Citrus Research Institute, Southwest University, Beibei, Chongqing 400715, China; Integrative Science Center of Germplasm Creation in Western China, Southwest University, Beibei, Chongqing 400715, China; National Citrus Engineering Research Center, Citrus Research Institute, Southwest University, Beibei, Chongqing 400715, China; Integrative Science Center of Germplasm Creation in Western China, Southwest University, Beibei, Chongqing 400715, China; National Citrus Engineering Research Center, Citrus Research Institute, Southwest University, Beibei, Chongqing 400715, China; Integrative Science Center of Germplasm Creation in Western China, Southwest University, Beibei, Chongqing 400715, China; National Citrus Engineering Research Center, Citrus Research Institute, Southwest University, Beibei, Chongqing 400715, China; Integrative Science Center of Germplasm Creation in Western China, Southwest University, Beibei, Chongqing 400715, China

## Abstract

Citrus Huanglongbing (HLB) is the most destructive disease in citriculture, mainly caused by *Candidatus* Liberibacter asiaticus (*C*Las). However, the immune response of citrus to *C*Las at the cellular level remains to be elucidated. In this study, the first single-cell atlas of rough lemon (*Citrus jambhiri* Lush.) root apexes were generated using single-nucleus RNA sequencing at 20 weeks postinoculation with *C*Las. According to gene expression patterns, the single-cell atlas was partitioned into 20 transcriptionally distinct clusters, and five cell types were identified within these clusters. A significant number of defense-related genes were co-upregulated across the five cell types following *C*Las infection, whereas genes involved in signal transduction pathways, such as tubulin beta-6 chain (*TUBB1*) and the phospholipase D alpha 1 (*PLD1*), were concurrently downregulated. Based on pseudotime trajectory analysis, the key pathways and genes involved in the coordination of cell differentiation and resistance in citrus under *C*Las infection were characterized. Following *C*Las infection, the development of phloem cells was significantly delayed, and the differentiation of cambium cells into xylem cells was evident. The expression of genes associated with lignin synthesis was significantly upregulated in these cells. The reduction in phloem cell differentiation and the enhanced differentiation of cambium cells into defense-related xylem cells may represent the primary vascular immune mechanisms exhibited by citrus plants in response to *C*Las infection. Additionally, DNA-binding one zinc finger transcription factor *DOF2.4* was found to potentially serve dual roles in regulating vascular cell development and inducing plant resistance against *C*Las. In conclusion, this study collectively provides insights into the cellular innate immunity responses of citrus to *C*Las infection. These findings hold significant implications for the sustainable development of citriculture amidst the ongoing global HLB epidemic, and offer novel insights into vascular immunity and plant defense responses.

## Introduction

Citrus is one of the major fruit crops worldwide due to its high economic and nutritional value. Huanglongbing (HLB) is the most devastating disease of citrus, mainly caused by noncultured, phloem-limited, and Gram-negative *Candidatus* Liberibacter asiaticus (*C*Las) [1, 2]. HLB-infected trees display symptoms such as stunted growth, blotchy mottle leaves, roots necrosis, and often produce fruit with discoloration starting at the peduncular end [1]. The formation of these symptoms is mainly attributed to the excessive accumulation of callose, followed by phloem blockage and collapse [3], which disrupts the translocation of photosynthetic products from source to sink [3, 4], resulting in altered root physiology and a significant decrease in starch content [5]. Notably, *C*Las titers are frequently detected in the roots, leading to a substantial reduction in root density, even in asymptomatic trees. This suggests that *C*Las initially targets the roots, where it replicates and damages the host root system [6]. However, a solid scientific explanation for this characteristic of *C*Las remains lacking.

Currently, significant progress has been made in understanding the pathogenesis of HLB caused by *C*Las, with several secreted effector proteins implicated in the disease process [7–9]. For instance, SDE19 (SEC-delivered effector 19) targets and destabilizes the citrus guanine nucleotide exchange factor SEC12, thereby inhibiting the secretion of apoplastic defense-related proteins, including PR1, P69B, GmGIP1, and RCR3 [7]. Transgenic sweet oranges expressing *C*Las0185 exhibit heightened sensitivity to *C*Las, which is attributable to *C*Las0185 targeting of methionine sulfoxide reductase B1 (*Cs*MsrB1). This interaction disrupts the redox balance and reduces the expression of ascorbate peroxidase 1 (*Cs*APX1) under oxidative stress [9]. Additionally, transgenic citrus plants expressing *C*Las4425 show reduced salicylic acid (SA) levels and downregulated expression of SA signaling-related genes [8]. Although these effectors have distinct functions, they collectively aim to suppress host immune responses and facilitate *C*Las proliferation.

Evolutionarily, plants have developed pathogen-associated molecular pattern-triggered immunity (PTI) and effector-triggered immunity (ETI) [10]. Plant immunity is marked by the triggering of a kinase signaling cascade, transcriptional reprogramming, and a burst of reactive oxygen species (ROS). It also includes the activation of defense-related genes, enhanced hormone signaling, and the synthesis of numerous antimicrobial secondary metabolites [10, 11]. Like other organisms, citrus plants mount a robust innate immunity response to *C*Las infection [12–15]. Transgenic citrus plants overexpressing *SABP2* (SA binding protein 2) exhibit increased SA content, reduced HLB symptom severity, and lower *C*Las titers [16, 17]. The immune response is further enhanced by both endogenous and exogenous methyl salicylate, which is regulated by the O-methyltransferase cascade initiated by miR2977-SAMT (SA regulatory code) [18]. *C*Las infection induces the upregulation of *CsABI5*, a key ABA signaling regulator in citrus leaves, leading to enhanced callose synthase activity [19]. However, elevated levels of SA and ABA are associated with a significant reduction in auxin content, leading to a notable decrease in unit cell numbers in diseased leaves [15]. This phenomenon may result from the reallocation of energy resources to defense mechanisms in response to *C*Las infection, potentially suppressing growth.

Alterations in hormone signaling have been shown to disrupt the overall transcriptional homeostasis of citrus, leading to phloem hyperplasia [14]. Indeed, differential expression of sieve element occlusion (SEO) and wall-associated receptor kinase-like 15 (*WAKL15*) has been documented in *C*Las-infected citrus plants [13]. Comparative transcriptomic analyses suggest that phloem regeneration and reduced phloem blockage contribute to the resistance of lemons to *C*Las infection [20]. Secondary metabolites also play a crucial role in citrus resistance to *C*Las infection [21, 22]. In HLB-tolerant citrus varieties, flavonoid content remains high, and *C*Las significantly increases the release of β-caryophyllene, β-ocimene, and nerolidol glucoside [21]. In contrast, flavonoid biosynthesis is impaired in *C*Las-infected citrus, while elevated levels of certain polyamines, including feruloylutamide, are linked to reduced disease severity [22]. Moreover, *C*Las is capable of converting the precursor ferulic acid, thereby impeding the biosynthesis of ferulic acid and downstream flavonoids [22].

**Figure 1:**
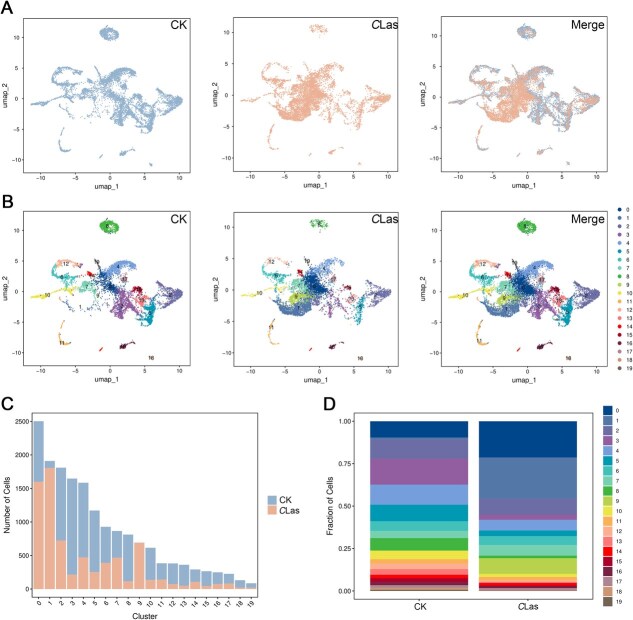
Single-nucleus RNA-sequencing of rough lemon at 20 weeks post-inoculation with *C*Las. (A) UMAP visualization of snRNA databases of citrus root at the infected and Mock condition. (B) UMAP visualization of the 20 cell clusters identified by unsupervised clustering analysis. (C) Cell numbers and (D) percentage of cells numbers allocated in the 20 cell clusters for two biological replicates of *C*Las-infection and CK samples.

However, current studies illustrate the collective outcomes of diverse cell types within a plant, often overlooking the heterogeneity in gene expression among individual cells. In reality, individual cell types exhibit varied responses to the stress induced by pathogenic microorganisms. Single-cell RNA sequencing (scRNA-seq) is a recently developed and sophisticated technique that can precisely characterize the gene expression patterns of thousands of individual cells from a single sample [23–25]. In recent years, this technique has been utilized to study horticulturally important plants [23–26]. However, the presence of a cell wall in plant cells poses significant difficulties in their isolation and manipulation, which hinders the implementation of scRNA-seq in mature and lignified plant organs [27]. Therefore, the advent of single-nucleus RNA sequencing (snRNA-seq) offers a more feasible alternative for plant research [27].

Rough lemon (*C*. *jambhiri* Lush.) is a widely used rootstock. Our preliminary work found that the overexpression of the effectors *C*Las1775 and *C*Las0485 caused significant accumulation of callus and hydrogen peroxide (H_2_O_2_). Consequently, we selected this material as the subject of our research on cellular immune responses. To understand how the transcriptional identities of each cell type in vascular tissue respond to *C*Las infection, this study generated the first single-nucleus transcriptomic atlas of *C*Las-infected rough lemon. Five cell types, including phloem, xylem, and cambium cells, were identified from 20 cell clusters. Furthermore, based on the pseudotime trajectory analysis, we characterized key pathways and genes involved in coordinating cell differentiation and resistance in citrus under *C*Las infection. In brief, this research provides an example of applying snRNA-seq analysis to study the complex regulation of citrus responses to *C*Las infection.

## Results

### snRNA-Seq characterizing the gene expression profiles of citrus roots apex cells

The present study generated single-nucleus transcriptomic atlases focusing on rough lemon roots infected with *C*Las (referred to as the *C*Las group) and healthy roots (CK group). A total of 7483 and 9431 single cells from two replicates were used in further analysis after quality control measures were applied to the raw data (Supplementary Table 1). Subsequent snRNA-seq analysis revealed an average of ~1130 genes and 1500 unique molecular identifiers (UMIs) per cell (Supplementary Table 1). The proportion of mitochondrial genes remained below the 5% threshold (Supplementary Fig. 1; Supplementary Table 1), indicating the data were reliable [28]. Uniform manifold approximation and projection (UMAP) analysis showed substantial overlap between cell populations from the *C*Las and CK groups (Fig. 1A).

Subsequent classification of these cells into 20 major clusters (Fig. 1B). Most of the top-5 marker genes were uniquely enriched in each corresponding cluster (Supplementary Fig. 2). The clusters were labeled from 0 to 19 based on cell abundance, from highest to lowest. Notably, both Cluster 1 and Cluster 9 showed a significant increase in cell count in the *C*Las group (Fig. 1C). The cell number proportion of Cluster 0 in CK was 9.57%, whereas in the *C*Las group, it was 21.38%. Cluster 1 exhibited a minimal proportion in CK (1.1%), but a substantial increase to 24.13% in the *C*Las group. Similarly, Cluster 9 increased from 0.03% in CK to 9.21% in the CLas group. Conversely, Clusters 3 and 5 showed pronounced decreases in relative proportions, from 15.18% to 2.87% and from 9.73% to 3.38%, respectively (Fig. 1D). These findings suggest that *C*Las infection alters the differentiation landscape of citrus root cells.

### Identification of cell types

**Figure 2:**
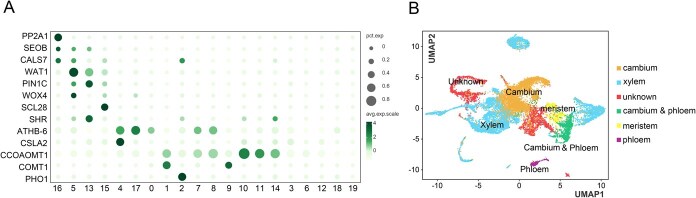
Cell types identified in citrus root. (A) Proportions and average expression levels (scaled) of selected marker genes. (B) UMAP visualization of citrus cell types including cambium cells, xylem cells, cambium and phloem cells, phloem cells, meristem cells, and an unknown cell type. Each dot represents a single cell. The colors of the dots correspond to cell clusters.

In the absence of well-established marker genes for citrus, the cell types of these clusters were annotated using homologous genes of previously reported markers (Supplementary Table 2) [29, 30]. For instance, phloem protein 2 (*PP2*) exhibits lectin properties and can be isolated from the phloem sap [31]. WUSCHEL-related homeobox (*WOX4*) plays a crucial role in maintaining the cambium by enhancing proliferation [32]. Glucomannan 4-beta-mannosyltransferase (*CSLA*) plays a role in cambium activities and secondary xylem formation [33]. The 4-coumarate coenzyme A ligase (*4CL*) and caffeoyl coenzyme A-3-O-methyltransferase (*CCOAOMT*) are essential for xylem-specific expression [34]. PHOSPHATE 1 (*PHO1*) is known to be expressed in the root pericycle and xylem parenchyma cells [35]. These marker genes served as references to assign cell clusters to each main cell type (Fig. 2A).

This included cambium (Clusters 0, 4, and 17), xylem (Clusters 1, 2, 7–11, and 14), cambium and phloem (Clusters 5), meristem (Clusters 13 and 15), and phloem (Clusters 16) cells. Clusters 3, 6, 12, 18, and 19 could not be assigned using the current marker set and were therefore classified as unknown cell types (Fig. 2B). This cell atlas represents a valuable resource for understanding the cellular responses to *C*Las infection in citrus roots.

### Citrus mounts a broad cellular immune response during *C*Las infection

**Figure 3:**
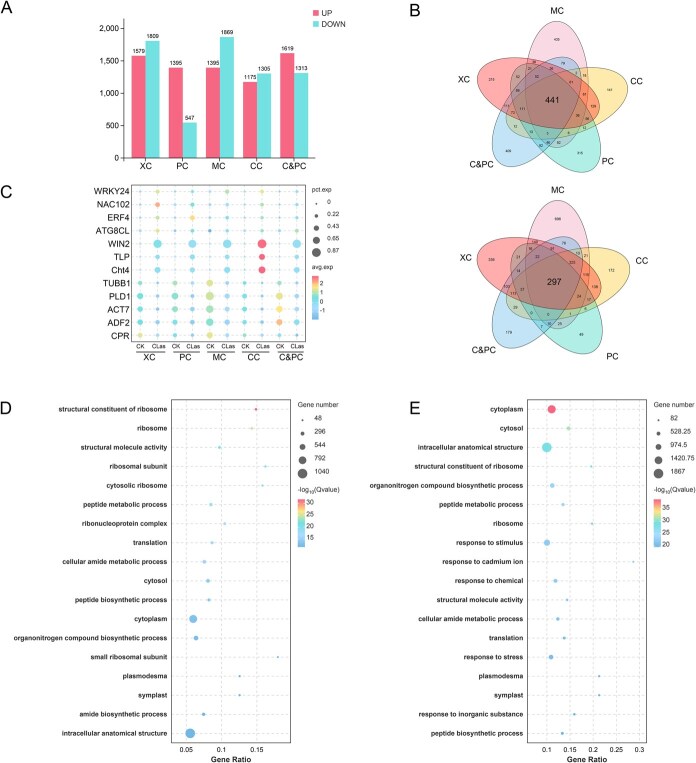
Differentially expressed genes (DEGs) in response to *C*Las infection in different cell types. (A) The bar plot shows the number of up and down DEGs at infected group for each cell type of *C*Las group, compare to CK. (B) Venn diagram of the number of up- and downregulated DEGs between different cell types. (C) Expression patterns of *WRKY24*, *NAC102*, *ERF4*, *ATG8CL*, *WIN2*, *TLP*, *Cht4*, *TUBB1*, *PLD1*, *ACT7*, *ADF2,* and *CPR* in five cell types. Gene Ontology (GO) enrichment of DEGs across phloem (D) and xylem (E) cells.

Differentially expressed genes (DEGs) between the *C*Las and CK groups were identified for each cell type using thresholds of |log2FC| ≥ 0.36 and *P* ≤ 0.05. In total, 14 006 DEGs were detected in the *C*Las group compared to the CK. As depicted in Fig. 3A, 3388 DEGs (1579 up, 1809 down), 1942 DEGs (1395 up, 547 down), 3264 DEGs (1395 up, 1869 down), 2480 DEGs (1175 up, 1305 down) and 2932 DEGs (1619 up, 1313 down) were detected in xylem cells, phloem cells, phloem and cambium cells, cambium cells and meristem cells, respectively. Among these, 441 upregulated and 297 downregulated DEGs were commonly shared across all five cell types (Fig. 3B). The variation in DEG numbers across cell types highlights the heterogeneity in cellular responses to *C*Las infection, potentially reflecting distinct defense or susceptibility mechanisms.

The *C*Las group exhibited a coordinated upregulation of multiple transcription factors, including *ERF4*, *WRKY24*, and *NAC102* (Fig. 3C), which are known to regulate plant physiological processes [36]. Additionally, autophagy-related gene (*ATG8CL*), pathogenesis-related protein (*WIN2*), thaumatin-like protein (*TLP*), and chitin (*Cht4*), were significantly upregulated (Fig. 3C). The specific degradation of *Cs*ATG8 family has been shown to inhibit autophagy, negatively regulate citrus immunity, and promote the proliferation of *C*Las [37]. Pathogenesis-related proteins, thaumatin-like proteins, and chitin are implicated in plant defense mechanisms [38]. Conversely, genes associated with signal transduction pathways, such as the tubulin beta-6 chain (*TUBB1*), the phospholipase D alpha 1 (*PLD1*), the actin-7 (*ACT7*), actin-depolymerizing factor (*ADF2*), and the NADPH-cytochrome P450 reductase (*CPR*), were co-downregulated in the *C*Las group (Fig. 3C) [39, 40]. These findings indicate that *C*Las activates downstream immune-related gene expression while simultaneously weakening signaling components involving cell membranes, microfilaments, and microtubules.

To gain deeper insights into the functional characteristics of DEGs across distinct cell types, a Gene Ontology (GO) enrichment analysis was conducted. The results revealed that DEGs were predominantly enriched in ‘cellular process’ and ‘metabolic process’ within the category of biological process. In terms of molecular function, ‘binding’ and ‘catalytic activity’ were the most represented. With respect to cellular components, the DEGs were primarily concentrated in ‘cellular anatomical entity’ and ‘protein-containing complex’ (Supplementary Fig. 3). DEGs of all cell types demonstrated significant enrichment in structural constituents of ribosomes, cytoplasm, and cytosol (Fig. 3D and E; Supplementary Fig. 4A–C). Notably, xylem cells showed significant enrichment in stress resistance-related GO terms, including ‘response to stimulus,’ ‘response to cadmium ion,’ ‘response to chemical,’ and ‘response to stress’ (Fig. 3E). These findings suggest that xylem cells may play a pivotal role in defensive responses against *C*Las infection.

### *C*Las infection suppresses phloem cells differentiation

**Figure 4:**
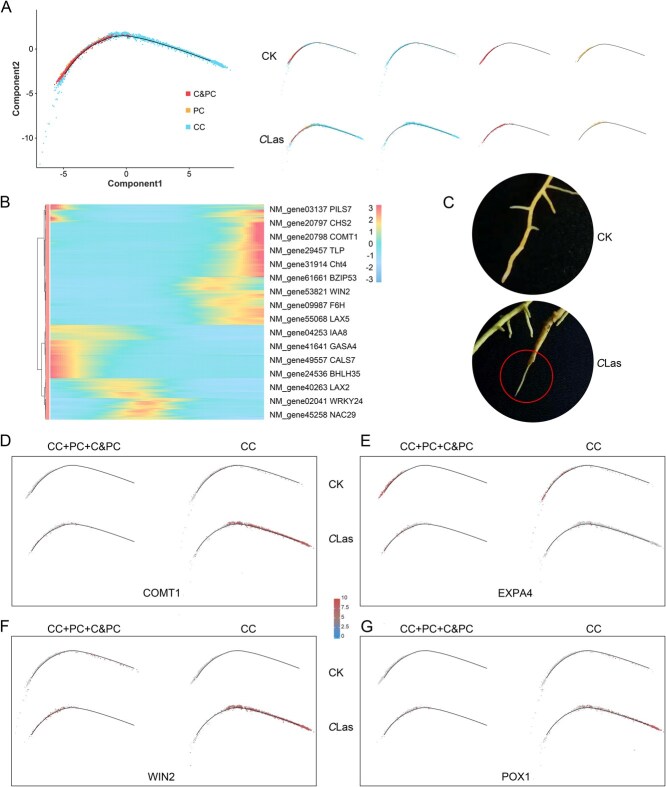
Phloem cells exhibit a delay in development under *C*Las infection. (A) Pseudotime trajectory of phloem cells inferred by Monocle 2 from right to left, representing all cells and different groups. Each dot indicates a single cell. (B) Smoothed expression heatmap of the top 500 altered genes of phloem cell along the differentiation trajectory from *C*Las group and CK. (C) The root tip cortex of citrus infected with *C*Las is easily exfoliated. Pseudotime trajectory of phloem cells inferred by Monocle 2 from right to left, representing all cells and different groups. Each dot indicates a single cell. Distribution of (D) *COMT1*, (E) *EXPA4*, (F) *WIN2,* and (G) *POX1* in phloem cell type on the pseudotime trajectory.

Phloem cells are the main place of survival of *C*Las. To explore the developmental trajectories from cambium to the phloem tissues, this study utilized cambium cells (Clusters 0, 4, and 17), cambium and phloem cells (Cluster 5), and phloem cells (Cluster 16), reconstructing a pseudotime trajectory consisting of a single track (Fig. 4A). Along the pseudotime trajectory, a progressive transition from cambium to phloem cells was observed, spanning from the right to the left end. The majority of cells in the CK group were more concentrated at the left of the trajectory, whereas the *C*Las group displayed a significantly delayed progression (Fig. 4A). These findings suggest that *C*Las infection inhibits the differentiation of cambium cells into phloem cells.

Subsequently, a comparative analysis was conducted on the top 500 genes along the pseudotime trajectory in the *C*Las group and CK (Fig. 4B). The results indicated a noteworthy phenomenon: genes linked to secondary metabolism and development exhibited significantly higher expression in the *C*Las group, whereas auxin-related genes were markedly downregulated (Fig. 4B; Supplementary Table 3). Specifically, several genes involved in flavonoid and lignin biosynthesis, including chalcone synthase (*CHS2*), caffeic acid 3-O-methyltransferase (*COMT1*), and feruloyl CoA ortho-hydroxylase (*F6H*), exhibited elevated expression levels in the *C*Las group. In contrast, auxin transporter-like protein 5 (*LAX5*) and auxin-responsive protein (*IAA27*) showed reduced expression levels (Fig. 4B). Furthermore, multiple transcription factors associated with abiotic stress response, including *WRKY24*, *NAC29*, *BHLH35*, and *BZIP53*, were highly enriched. Notably, *C*Las-infected roots showed increased susceptibility to root epidermis shedding. This phenomenon involves a complex interplay between callose-induced phloem necrosis and the inhibition of phloem cell development (Fig. 4C).

The pseudotime trajectory analysis further substantiated this finding. For instance, the xylem development gene *COMT1* exhibited elevated expression levels in cambium cells of the *C*Las group (Fig. 4D). Conversely, the expansin-related gene *EXPA*, a pivotal component of cell wall loosening, exhibited significantly reduced expression in both phloem and cambium cells of the *C*Las group (Fig. 4E). Similarly, the wound-induced protein WIN2, a positive regulator of tracheary element formation, vascular reconnection, and pathogen resistance, was highly expressed in cambium cells of the *C*Las group (Fig. 4F). In addition, *POX1*, which encodes proline dehydrogenase and plays a vital role in osmotic regulation, was also upregulated in cambium cells in the *C*Las group (Fig. 4G). These findings suggest that *C*Las infection suppresses the differentiation of cambium cells into phloem cells, while promoting their differentiation toward xylem-related cells, alongside enhanced expression of defense-related genes.

### *C*Las infection promotes specific differentiation of xylem defense-related cells

**Figure 5:**
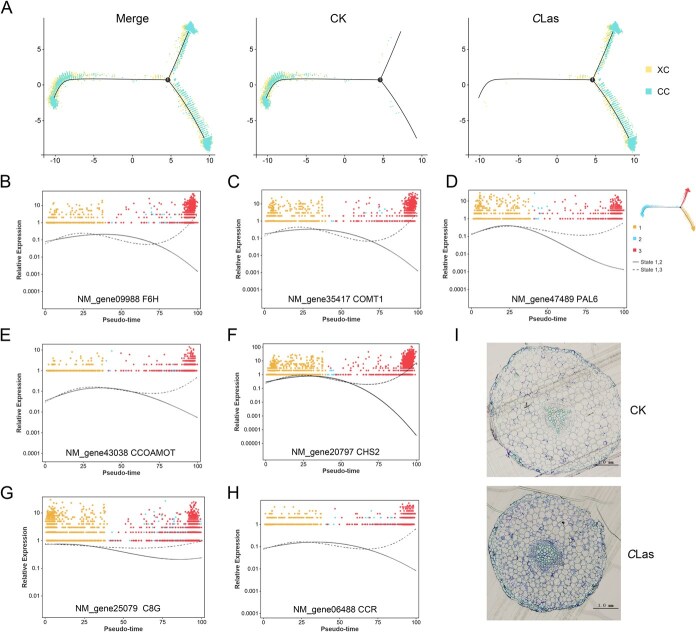
Xylem cells differentiate into defense related cells under *C*Las infection. (A) A continuous differentiation trajectory from cambium cell to xylem cell was obtained using Monocle 2. Each dot indicates a single cell. (B) *F6H*, (C) *COMT1*, (D) *PAL6*, (E) *CCOAMOT*, (F) *CHS2*, (G) *C8G* and (H) *CCR* expression dynamics over the differentiation trajectory in xylem cells. A dot represents a cell, and the colors denote the cell-type cluster, different branches represent different cell states. (I) The accumulation of lignin was detected by toluidine blue staining.

To further investigate DEGs identified in xylem cells during *C*Las infection, pseudotime analysis was performed. Cambium and xylem cells in the CK group continued to differentiate towards the left, whereas those cells in the *C*Las group shifted upward and progressed toward the upper right (Fig. 5A). These observations suggest that *C*Las infection alters the developmental trajectory and differentiation pattern of xylem cells.

Subsequently, the most significant expression changes among the top 500 differentiation fate-associated DEGs across pseudotime were analyzed (Fig. 5B; Supplementary Table 4). A notable number of genes involved in flavonoid and lignin biosynthesis pathways were highly expressed in the *C*Las group, including *F6H*, *COMT1*, *CHS2*, *PAL6* (Phenylalanine ammonia-lyase), *CCOAOMT1* (caffeoyl-CoA O-methyltransferase), *C8G* (coumarin 8-geranyltransferase), and *CCR* (cinnamoyl-CoA reductase) (Fig. 5B). Given toluidine blue's ability to stain lignin with high specificity [41], this method was employed to detect lignin accumulation. Staining results showed that most cells in the *C*Las group exhibited blue and purple coloration, suggesting enhanced lignin accumulation. Importantly, this increase was not restricted to xylem cells (Fig. 5C). These findings suggest that to defend against *C*Las infection, cells synthesize more secondary metabolites such as lignin.

In addition, the marked expansion of Clusters 1 and 9 following *C*Las infection suggested that xylem cells may undergo a specific pattern of differentiation. To further confirm this hypothesis, GO and KEGG enrichment analyses were performed (Supplementary Fig. 5). A large proportion of the enriched GO terms in these cell types were associated with stress responses (Supplementary Fig. 5A and B). For example, DEGs involved in ‘response to stimulus’, ‘response to chemical’, ‘response to organic substance’, ‘response to cadmium ion’, ‘response to stress’, ‘response to inorganic substance’, and ‘response to metal ion’ were enriched in Clusters 1 and 9. Furthermore, KEGG analysis revealed co-enrichment of upregulated genes in pathways such as ubiquitin mediated proteolysis (ko:04120), phagosome (ko:04145), citrate cycle (ko:00020), carbon metabolism (ko:01200), oxidative phosphorylation (ko:00190), biosynthesis of amino acids (ko:01230), glyoxylate and dicarboxylate metabolism (ko:00630), pyruvate metabolism (ko:00620) (Supplementary Fig. 5C and D). These findings indicate that Clusters 1 and 9 may represent the primary xylem cell populations involved in defense mechanisms against *C*Las infection.

### Development or defense depends on a combination of different hormones

**Figure 6:**
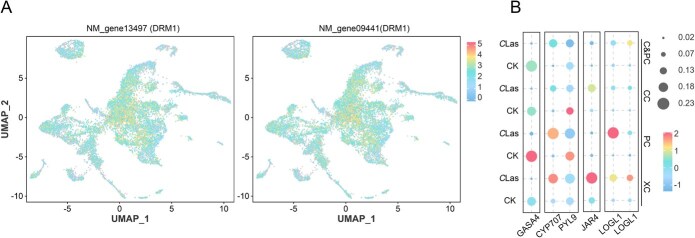
Expression patterns of hormones related gene under *C*Las infection. (A) UMAP visualization of two DRM1 expressions (NM_gene13497 and NM_gene09441). (B) Expression patterns of *GASA4*, *CYP707*, *PYL9*, *JAR4*, and *LOGL1* in four cell types.

The differentiation of phloem and xylem cells in vascular plants is regulated by key hormones including auxin, gibberellin (GA), cytokinin, and jasmonic acid (JA) [42–44]. Therefore, an analysis was performed on the expression levels of hormones related genes across different cell types (Fig. 6; Supplementary Fig. 6).

Two auxin-repressed genes (*DRM1*) showed significantly increased expression in phloem and cambial cells in the *C*Las group and were broadly expressed across all cell types (Fig. 6A; Supplementary Fig. 6). Furthermore, several auxin-related genes in the CK group, including *ARF5*, *IAA8*, *LAX5*, and *PIN6*, showed elevated expression specifically in cambium and phloem cells; however, their expression levels were markedly reduced following *C*Las infection (Supplementary Fig. 6). GA has been reported to modulate auxin activity during cambium differentiation [44]. This research found that a gibberellin-regulated gene (*GASA4*) was downregulated during *C*Las infection (Fig. 6B). Similarly, an ABA receptor gene (*PYL9*), involved in the ABA pathway, also showed reduced expression (Fig. 6B). Conversely, *CYP707* (ABA 8′-hydroxylase) in the ABA pathway, *JAR4* (JA-amido synthetase) in the JA pathway, and the two cytokinin riboside 5′-monophosphate phosphoribohydrolase gene (*LOG1*) exhibited significant upregulation in the *C*Las group (Fig. 6B).

### Overexpression of *CjDOF2.4* regulates root vascular tissue differentiation and promotes H_2_O_2_ accumulation

DNA-binding one zinc finger (DOF) transcription factors play a pivotal role in the development of plant vascular tissue and in plant defense responses against pathogens [45, 46]. This study found that *DOF2.4* underwent expression changes in response to *C*Las infection by integrating RNA-seq data from previously published studies by Peng *et al*. and Liu *et al*. (Supplementary Table 5 and Supplementary Fig. 7A) [14, 47]. Given the use of different citrus species across the three transcriptomic datasets (namely, wild mandarin (*C. reticulata*) in Peng *et al*., kaffir lime (*C. hystrix*) in Liu *et al*., and rough lemon in the present study), this study analyzed the expression of *CjDOF2.4* in *C*Las-infected rough lemon roots using RT-qPCR. The results revealed that *CjDOF2.4* was upregulated in *C*Las-infected roots (Supplementary Fig. 7B).

**Figure 7:**
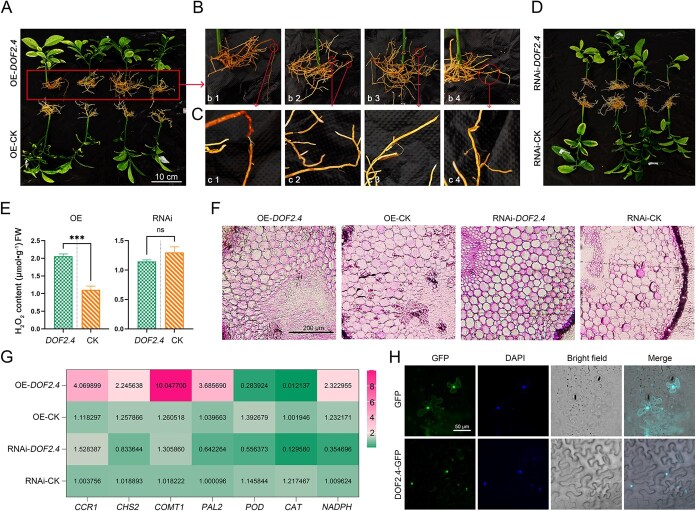
Analysis of rough Lemon Hairy Root Transformation of *CjDOF2.4*. (A) The roots overexpression *CjDOF2.4*. (B) is a close-up of the root in A. (C) is a close-up of the symptomatic area at the root in B. (D) The *CjDOF2.4*-RNAi roots. (E) Determination of H_2_O_2_ content. (F) Root tissue section observation. (G) RT-qPCR analysis of *CCR1*, *CHS2*, *COMT1*, *PAL2*, *POD*, *CAT*, and *NADPH*. *GADPH* was used as an internal reference control gene. (H) Subcellular localization of *Cj*DOF2.4 in tobacco leaf epidermal cells by *Agrobacterium*-mediated transformation. The nucleus was stained with DAPI. Bar = 50 μm. Standard deviations were indicated by error bars. The asterisks indicated significant differences using Student’s *t*-test. (ns *P* ≥ 0.05, ^***^P < 0.001).

To further investigate the role of *CjDOF2.4* in the response to *C*Las infection, this study generated transgenic hairy roots overexpressing *CjDOF2.4* (OE-*CjDOF2.4*) and hairy roots with *CjDOF2.4* expression suppressed via RNA-interference (RNAi-*CjDOF2.4*) (Fig. 7A–D). OE-*CjDOF2.4* roots exhibited root epidermal shedding and a browner external appearance compared to the control (Fig. 7A–C). However, RNAi-*CjDOF2.4* roots showed no significant morphological differences from the control (Fig. 7D).

Given the observed root epidermal shedding and browning in this experiment, it was hypothesized that these phenotypes might result from oxidative damage. To evaluate this hypothesis, the present study quantified H₂O₂ content in the transgenic roots. As anticipated, OE-*CjDOF2.4* roots exhibited higher levels of H_2_O_2_ compared to the control (Fig. 7E). To further investigate the underlying cellular changes, histological sectioning of the transgenic roots was performed. Cells in the OE-*CjDOF2.4* roots displayed irregular growth patterns, in contrast to those in the control or RNAi-*CjDOF2.4* roots (Fig. 7F). Subsequent qPCR analysis revealed that *PAL2* (a gene involved in the phenylpropanoid metabolic pathway), *CCR1* (a gene related to lignin synthesis), *COMT1*, *CHS2* (a gene related to flavonoid synthesis), and *NADPH* (a gene related to H_2_O_2_ synthesis) were all upregulated in the OE-*CjDOF2.4* roots (Fig. 7G). Concurrently, the expression of *POD* and *CAT*, which decompose H_2_O_2_ into H_2_O, was suppressed (Fig. 7G). In the RNAi-*CjDOF2.4* roots, the expression of *CCR1* and *COMT1* demonstrated no significant alterations (Fig. 7G); however, *PAL2* and *CHS2* exhibited a slight decreasing trend. Additionally, the expression of *POD* and *CAT* was also downregulated in the RNAi-*CjDOF2.4* roots, whereas *NADPH* expression showed a contrasting trend compared to that observed in the OE-*CjDOF2.4* roots (Fig. 7G). Additionally, subcellular localization studies revealed that *Cj*DOF2.4 performs its function within the nucleus (Fig. 7H).

## Discussion

In the course of extended interaction with pathogens, plants have developed a sophisticated and multilayered innate immune system that serves as a critical defense mechanism against pathogen invasion [10, 11]. The phloem-specific parasitism of *C*Las in citrus instigated our curiosity regarding how different cell types respond to infection. Consequently, snRNA-seq was employed to explore cell type–specific gene expression changes following *C*Las infection.

The snRNA-seq data were initially divided into 20 distinct cell clusters based on differential gene expression patterns (Fig. 1B). These cell clusters were further categorized into five major groups, namely phloem cells, xylem cells, cambium cells, cambium and phloem cells, and meristem cells, according to the known homologous marker genes (Fig. 2A and B). Analysis of DEGs revealed that defense-related genes were upregulated across all cell types (Fig. 3A–E; Supplementary Fig. 3 and Supplementary Fig. 4). This result was consistent with the findings from previous studies that have conducted extensive sequencing on *C*Las-infected citrus leaves [12–15]. For instance, Gao *et al*. conducted an RNA-seq study using leaves from HLB-tolerant *C. limon* and *C. maxim* as materials. The analysis reveals that during the initial phases of *C. limon* and *C. maxim* infection (12 weeks post inoculation), genes associated with PTI, cell wall-associated immunity, endochitinase, and phenylpropanoid are significantly activated [12]. These observations collectively suggest that the citrus innate immune response to *C*Las infection is mediated through a conserved and coordinated activation of defense signaling pathways.

However, traditional omics approaches primarily aim to characterize plant responses to pathogens at the whole-tissue level, which obscures the unique features of individual cell populations [26]. In the subsequent GO enrichment analysis, the majority of defense-related DEGs were predominantly enriched in xylem cells, while the phloem cells showed limited enrichment (Fig. 3D and E). This unexpected result prompted further investigation through a pseudotime trajectory analysis of cambium and phloem cells, which revealed that *C*Las infection disrupted the transition of cambium cells into phloem cells (Fig. 4A). Additionally, cambium cells exhibited significant upregulation of xylem-specific genes under infection (Fig. 4A), a phenomenon that conventional transcriptomics cannot resolve. Indeed, traditional transcriptomic studies report that DEGs encoding immune receptors are downregulated in *C. reticulata* leaves infected with *C*Las, while DEGs involved in signal transduction and plant-pathogen interactions are upregulated in *C*Las-infected bark [47]. These discrepancies across tissue types may stem from differences in cellular composition, highlighting the necessity for cell-type-specific analyses to accurately interpret plant immune responses.

Furthermore, our dates showed a decline in phloem cell numbers (Fig. 1D). This finding suggests that citrus suppresses phloem development under *C*Las infection and instead differentiates into xylem cells, which exhibit enhanced resistance (Fig. 4A and Fig. 5A). Pseudotime analysis of cambium and xylem cells further demonstrated distinct differentiation patterns in the *C*Las group compared to the CK group (Fig. 5A). Notably, genes associated with secondary metabolism, particularly those involved in phenylpropanoid metabolism and lignin biosynthesis, were significantly upregulated in the *C*Las group (Fig. 5B–H). Consistent with this, toluidine blue staining demonstrated that nearly all cells in the *C*Las group exhibited a substantial accumulation of lignin (Fig. 5I). Interestingly, cells 1 and 9 were nearly absent under normal growth conditions (cell 1: 1.1%, cell 9: 0.03%) (Fig. 1C and D), but their proportions increased markedly following *C*Las infection. Importantly, not all toluidine blue-stained cells in the *C*Las group were xylem cells, suggesting that cells 1 and 9 may have undergone lignification post-infection, potentially contributing to enhanced resistance (Fig. 6A–D). These findings strongly indicate that the phenylpropanoid metabolic pathway, particularly lignin biosynthesis, represents a conserved defense mechanism against *C*Las infection, primarily active in xylem or lignified cells.

The induction of lignin biosynthesis plays a critical role in the establishment of vascular-specific immunity [48]. Research has demonstrated that the thickening and lignification of cell walls in *Pinus massoniana* can mitigate damage to vascular tissue caused by nematodes when they resist the infection of pine wood nematodes [49]. Furthermore, certain lignin synthesis enzymes are also involved in the biosynthesis of flavonoids, indoles, terpenoids/bibenzenes, alkaloids, and coumarins [50]. Nonetheless, this phenomenon appears to be detrimental to the long-term growth of citrus. Ectopic lignin deposition in cells that are typically unlignified has been demonstrated to inhibit plant growth [51]. By integrating transcriptomic data from prior studies with snRNA-seq data from the present study [14, 47], we identified a transcription factor, *DOF2.4*, potentially implicated in vascular development and stress resistance under adverse environmental stress [45, 46]. Subsequent transgenic hairy root experiments revealed that the overexpression of *DOF2.4* induced the expression of key genes in the phenylpropanoid metabolic pathway, including genes related to lignin biosynthesis, leading to irregular root cell growth (Fig. 7F and G).

Furthermore, the results of this study demonstrated that the expression of genes associated with H_2_O_2_ hydrolysis was reduced by overexpressing *DOF2.4* (Fig. 7G). Consistent with this, transgenic roots overexpressing *DOF2.4* exhibited elevated H_2_O_2_ levels (Fig. 7E). Moreover, this study observed a noteworthy phenomenon: root epidermis peeling was evident in both *C*Las-infected roots and roots overexpressing *DOF2.4* (Fig. 4C and Fig. 7A–C). This observation likely reflects oxidative damage caused by excessive H_2_O_2_ accumulation. This finding was consistent with the results of a previous study on HLB [52]. Ma *et al*. conclude that citrus HLB is a host-immune disease caused by *C*Las, in which the production of ROS in response to *C*Las is localized in phloem cells, and it is followed by systemic cell death of companion and sieve element cells [52]. This evidence suggested that citrus faced two interconnected challenges during the immune response to *C*Las. Firstly, activation of the citrus's intrinsic immune system impeded the progression of the phloem. Secondly, this immune response may escalate into an excessive reaction, potentially causing cellular damage or death. Therefore, identifying the molecular mechanisms that balance citrus growth and innate immunity was crucial.

During vascular cambium cell differentiation, secondary xylem forms internally and secondary phloem develops externally [53]. Auxin is essential for cambium cell proliferation, and reduced auxin levels lead to decreased xylem cell division [42, 54]. Gibberellin treatment promotes an increase in auxin levels from the xylem side of the cambium toward the phloem. Xylem-side stem cell daughter preferentially differentiates into xylem cells, whereas reduced gibberellin levels favor the specification of phloem-side stem cells into phloem cells [44]. This study demonstrated that the expression of auxin inhibitor *DRM1* was significantly induced in phloem and xylem cells of the *C*Las group, while the expression of gibberellin-related gene *GASA4* was significantly downregulated (Fig. 6A and B; Supplementary Fig. 6). These results indicated that both phloem and xylem cells may be inhibited under *C*Las infection, but gibberellin-regulated phloem cell development was more severely inhibited. As demonstrated by Ma *et al*. [52], the application of exogenous GA has been shown to alleviate HLB to a certain extent.

Furthermore, the study found that several auxin-related genes, including *ARF5* and *LAX5*, were downregulated in *C*Las-infected cambium and phloem cells. Concurrently, *IAA13* and *IAA8* showed increased expression during this process (Supplementary Fig. 6). In the root vascular system of *Arabidopsis thaliana*, elevated cytokinin signaling in procambial cells has been shown to promote PIN-FORMED (PIN) auxin efflux proteins expression, which facilitates the transverse flow of auxin from procambial cells to meristem cells, contributing to protoxylem formation [55]. Our findings indicated that two genes associated with cytokinin (*LOG1*) were notably expressed in phloem and xylem cells of the *C*Las group (Fig. 6B), potentially linked to the differentiation of xylem defense-related cells in response to infection. The coordinated regulation of disparate hormonal signals may emerge as a pivotal factor in the determination of cellular differentiation and stress resilience under *C*Las infection.

## Conclusion

The present study demonstrates that citrus plants establish a multifaceted defensive system during *C*Las infection by modifying cell fate and initiating specific gene expression patterns. This includes the upregulation of genes encoding secondary metabolites, primarily in xylem cells, and the enhancement of cell lignification to inhibit phloem cell development. Moreover, our research identifies *DOF2.4* as a candidate gene with potential dual roles in regulating vascular cell development and enhancing plant resistance. This gene represents a promising target for enhancing the balance between growth and stress tolerance in citrus plants in response to *C*Las infection. Future studies will aim to elucidate the molecular mechanisms by which *DOF2.4*, in coordination with plant hormone signals, modulates citrus resistance to *C*Las. These findings hold significant implications for the sustainable development of citriculture amidst the ongoing global HLB epidemic, and offer novel insights into vascular immunity and plant defense responses.

## Materials and methods

### Plant material and sampling

In this study, 5-year-old rough lemon trees were prepared for snRNA-seq. The trees were cultivated in commercial citrus growing media (a mixture of peat, perlite, and vermiculite at a volume ratio of 3:1:1) in pots with dimensions of 25.5 × 26.5 × 30.0 cm. The greenhouse had an average temperature of 26°C. The trees infected with *C*Las via grafting and confirmed via PCR detection. The relevant primers are shown in Supplementary Table 6. Two rough lemon trees infected with *C*Las for 20 weeks and two healthy trees were selected as biological replicates for the experiment. At this stage, the *C*Las-infected plants exhibited no overt symptoms of HLB. Initially, the main roots of the plants (excluding the lateral roots) were collected, each measuring ~6 to 7 cm in length. These root segments were then divided into two segments: a 2-cm root apex and a 4- to 5-cm remaining part. Corresponding marks were made, and the root segments were rinsed with saline and snap-frozen in liquid nitrogen to minimize changes in gene expression. The remaining 4–5 cm of the main roots were then utilized for PCR testing to ascertain the infection status. Subsequently, ten root apexes corresponding to the 2 cm root apexes were collected and amalgamated as a sample for experimentation.

### snRNA library construction and sequencing

The technical service for the snRNA-seq was provided by Gene Denovo Biotechnology Co., Ltd. (Guangzhou, China), according to 10× Genomics (10× Genomics, USA). Crude nuclei were extracted from the root apexes, and a quality inspection was performed first. The crude nuclear suspension that met the quality requirements was then diluted to 1000 nuclei per milliliter. These nuclei were then mixed with enzymes and barcoded gel beads to form Gel Beads-In-Emulsions (GEMs). The GEMs were then transferred to a collection reservoir, where the embedded barcode sequences were revealed when the outer gel beads dissolved. These released sequences were enzymatically cleaved into fragments of ~200 to 300 bp, then poly-A tails, adapter sequences P5, and sequencing primers R1 were introduced, and finally, these products were collected to establish a sequencing library by using the Illumina NovaSeq X Plus. The cell and expression counts were then determined by incorporating 16-bp barcode information and 10-bp UMI information.

### Analysis of snRNA-seq data

The raw snRNA-seq data were first processed using Cell Ranger from 10x Genomics for filtering low-quality barcodes and UMIs (unique molecular identifiers), and aligned to the reference genome [20]. Subsequently, the UMI sequences were corrected to ensure the accuracy of data analysis, as the gene matrix was generated through UMI counting and barcoding. The corrected data were imported into Seurat (v3.1.1) for further analysis.

Doublet Finder (v2.0.3) was used to identify doublets, setting the threshold to a gene count of 200 to 8000 per cell, a mitochondrial gene percentage < 25%, and a UMI count below 50 000. Then, Harmony was employed to amalgamate the data, thus yielding the original data. Principal component analysis (PCA) was employed to reduce the dimensionality of the top variable genes. The FindAllMarkers function of the Seurat package was then utilized to cluster cells according to their gene expression profiles. UMAP analysis was then employed to visualize these clusters [56–58]. The top 10 DEGs were used as marker genes to distinguish different cell clusters. OmicShare (https://www.omicshare.com) was used for enrichment analysis, based on either the GO database (https://www.geneontology.org) or the KEGG database (https://www.kegg.jp). Cell types were annotated based on the expression of specific marker genes, and pseudotime trajectory analysis was performed using Monocle 2 [59].

### Phenotyping of cellular responses

The root apexes of rough lemon were meticulously sliced and observed. Specifically, 1 cm root apexes were excised and fixed in 40% FAA fixative solution (Biosharp, China) at a ratio of 1:10 (tissue: solution). The samples were dehydrated using acetone solution of varying concentrations. A low viscosity embedding kit (SPI, America) was then employed for the embedding. Then, ultrathin sections were cut using an EM UC7 ultramicrotome (Leica, Germany) to create the sections. Following this, the sections were stained with 0.1% toluidine blue for two minutes or 1% safranin-O for 30 minutes. Thereafter, the sections were rinsed with tap water and dried using a sheet oven. Finally, the sections were observed under an optical microscope.

### Real-time quantitative PCR (RT-qPCR) assay

To extract the total RNA from rough lemon roots, the kit named RNAiso Plus (Takara, Japan) was used in this study. The SYBR qPCR SuperMix Plus (Takara, Japan) was used for RT-qPCR. Set the following thermal cycling parameters on the CFX96TM Real-Time System (Bio-Rad, USA): with an initial hold at 95°C for 1 minute, followed by 45 cycles of 95°C for 20 seconds, 58°C for 20 seconds, and 72°C for 30 seconds. Then, followed by a melting curve program at 65°C to 95°C, raised gradually by 0.5°C every 5 seconds. The primers used for the RT-qPCR assays are enumerated in Supplementary Table 6. For analysis, the 2^−ΔΔCT^ method was utilized, and the *GAPDH* served as the internal reference control gene. Three technical replicates were set for each gene, and two biological replicates were performed.

### Gene cloning

The coding DNA sequences (CDS) of *CjDOF2.4* were cloned from rough lemon via RT-PCR. The relevant primers are shown in Supplementary Table 6.

### *A. rhizogenes* K599–mediated citrus root transformation

The full-length CDS of *CjDOF2.4* was cloned into the pLGN-OE vector (Kanamycin) containing a β-glucuronidase (GUS) gene to construct the overexpressing vector. An empty pLGN-OE vector was used as a control. Partial CDS fragments of *CjDOF2.4* (340–639 bp) were amplified and inserted into the pLGN-RNAi vector (Kanamycin) containing a *GUS* to construct the interference vector, respectively. The relevant primers are shown in Supplementary Table 6. *A. rhizogenes* K599 containing the corresponding overexpression and RNAi vectors were used to transform rough lemon plantlets. The root-mediated genetic transformation method was performed as previously described [60]. After 6 months, a histochemical GUS activity assay was then carried out using transient expression in the roots of rough lemon according to the manufacturer’s protocol in the GUS staining kit (Coolaber, China).

### Determination of H_2_O_2_ content

The levels of H_2_O_2_ were determined using the hydrogen peroxide measuring kit (Solarbio, China).

### Subcellular localization analysis

The CDS of *CjDOF2.4*, without a stop codon, was cloned into the pCV-GFP vector (Kanamycin). This generated a *Cj*DOF2.4-GFP fusion protein. An empty pCV-GFP vector was used as a control. The relevant primers are shown in Supplementary Table 6. The reconstructed vectors were then each changed into a strain of *A. tumefaciens* called GV3101 (Weidi, China). The *A. tumefaciens* harbouring the pCV-*Cj*DOF2.4-GFP vector was used to transform four-week-old *N. benthamiana* plants as previously described [61]. An inverted fluorescence microscope (IX-73, Olympus, Japan) was used to observe the presence of fluorescence signals in the transformed leaf tissue. The GFP was fluorescence excitation light wavelength 488 nm, and the DAPI was 405 nm.

### Statistical analysis

The statistical analyses and mapping software were performed using GraphPad Prism software version 10.3.0 (https://www.graphpad-prism.cn/). Student's *t* test was calculated for pairwise comparisons.

## Supplementary Material

Web_Material_uhaf265

## References

[1] Bové JM . Huanglongbing: a destructive, newly-emerging, century-old disease of citrus. J Plant Pathol. 2006;88:7–37

[2] Da Graça JV, Douhan GW, Halbert SE. et al. Huanglongbing: an overview of a complex pathosystem ravaging the world's citrus. J Integr Plant Biol. 2016;58:373–87 26466921 10.1111/jipb.12437

[3] Deng H, Achor D, Exteberria E. et al. Phloem regeneration is a mechanism for Huanglongbing-tolerance of "Bearss" lemon and "LB8-9" Sugar Belle® Mandarin. Front Plant Sci. 2019;10:277 30949186 10.3389/fpls.2019.00277 PMC6435995

[4] Fan J, Chen C, Yu Q. et al. Comparative transcriptional and anatomical analyses of tolerant rough lemon and susceptible sweet orange in response to *'Candidatus* Liberibacter asiaticus' infection. Mol Plant-Microbe Interact. 2012;25:1396–407 22809274 10.1094/MPMI-06-12-0150-R

[5] Etxeberria E, Gonzalez P, Achor D. et al. Anatomical distribution of abnormally high levels of starch in HLB-affected Valencia orange trees. Physiol Mol Plant Pathol. 2009;74:76–83

[6] Johnson EG, Wu J, Bright DB. et al. Association of ‘*Candidatus* Liberibacter asiaticus’ root infection, but not phloem plugging with root loss on huanglongbing-affected trees prior to appearance of foliar symptoms. Plant Pathol. 2014;63:290–8

[7] Huang G, Chang X, Hu Y. et al. SDE19, a SEC-dependent effector from *'Candidatus* Liberibacter asiaticus' suppresses plant immunity and targets Citrus sinensis Sec12 to interfere with vesicle trafficking. PLoS Pathog. 2024;20:e1012542 39255299 10.1371/journal.ppat.1012542 PMC11414923

[8] Zhang S, Wang X, He J. et al. A sec-dependent effector, CLIBASIA_04425, contributes to virulence in *'Candidatus* Liberibater asiaticus'. Front Plant Sci. 2023;14:1224736 37554557 10.3389/fpls.2023.1224736 PMC10405523

[9] Zhang S, Wang X, Zhao T. et al. Effector CLas0185 targets methionine sulphoxide reductase B1 of Citrus sinensis to promote multiplication of *'Candidatus* Liberibacter asiaticus' via enhancing enzymatic activity of ascorbate peroxidase 1. Mol Plant Pathol. 2024;25:e70002 39215961 10.1111/mpp.70002 PMC11365454

[10] Jones JDG, Dangl JL. The plant immune system. Nature. 2006;444:323–9 17108957 10.1038/nature05286

[11] Ngou BPM, Ding P, Jones JDG. Thirty years of resistance: zig-zag through the plant immune system. Plant Cell. 2022;34:1447–78 35167697 10.1093/plcell/koac041 PMC9048904

[12] Gao C, Li C, Li Z. et al. Comparative transcriptome profiling of susceptible and tolerant citrus species at early and late stage of infection by "*Candidatus* Liberibacter asiaticus". Front Plant Sci. 2023;14:1191029 37389294 10.3389/fpls.2023.1191029 PMC10301834

[13] Li R, Wang X, Hu Y. et al. Analysis of huanglongbing-associated RNA-seq data reveals disturbances in biological processes within Citrus spp. triggered by *Candidatus* Liberibacter asiaticus infection. Front Plant Sci. 2024;15:1388163 38660443 10.3389/fpls.2024.1388163 PMC11039969

[14] Liu Y, Dong L, Ran D. et al. A comparative analysis of three Rutaceae species reveals the multilayered mechanisms of citrus in response to Huanglongbing disease. J Plant Growth Regul. 2023;42:7564–79

[15] Neupane A, Shahzad F, Bernardini C. et al. Poor shoot and leaf growth in Huanglongbing-affected sweet orange is associated with increased investment in defenses. Front Plant Sci. 2023;14:1305815 38179481 10.3389/fpls.2023.1305815 PMC10766359

[16] Soares JM, Weber KC, Qiu W. et al. Overexpression of the salicylic acid binding protein 2 (SABP2) from tobacco enhances tolerance against Huanglongbing in transgenic citrus. Plant Cell Rep. 2022;41:2305–20 36107199 10.1007/s00299-022-02922-6

[17] Dong L, Chen S, Shang L. et al. Overexpressing CsSABP2 enhances tolerance to Huanglongbing and citrus canker in C. sinensis. Front. Plant Sci. 2024;15:1472155 10.3389/fpls.2024.1472155 PMC11493644 39439518

[18] Cheng B, Le X, Bilal MS. et al. Small RNAs contribute to citrus Huanglongbing tolerance by manipulating methyl salicylate signaling and exogenous methyl salicylate primes citrus groves from emerging infection. Plant J. 2023;116:1309–24 37614043 10.1111/tpj.16426

[19] Yao L, Guo X, Su J. et al. ABA-CsABI5-CsCalS11 module upregulates callose deposition of citrus infected with *Candidatus* Liberibacter asiaticus. Hortic Res. 2024;11:uhad276 38344648 10.1093/hr/uhad276 PMC10857934

[20] Bao Y, Zeng Z, Yao W. et al. A gap-free and haplotype-resolved lemon genome provides insights into flavor synthesis and huanglongbing (HLB) tolerance. Hortic Res. 2023;10:uhad020 37035858 10.1093/hr/uhad020 PMC10076211

[21] Wen H, Zhang S, Liu Y. et al. Screening universal stress-response terpenoids and their biosynthetic genes via volatile and transcriptomic profiling in citrus. J Agric Food Chem. 2024;72:351–62 38115585 10.1021/acs.jafc.3c06109

[22] Aksenov A, Blacutt A, Ginnan N. et al. Spatial chemistry of citrus reveals molecules bactericidal to *Candidatus* Liberibacter asiaticus. Sci Rep. 2024;14:20306 39218988 10.1038/s41598-024-70499-z PMC11366753

[23] Zhang J, Ahmad M, Gao H. Application of single-cell multi-omics approaches in horticulture research. Mol Hortic. 2023;3:18 37789394 10.1186/s43897-023-00067-y PMC10521458

[24] Guo Y, Chen X, Li J. et al. Single-cell RNA sequencing reveals a high-resolution cell atlas of petals in *Prunus mume* at different flowering development stages. Hortic Res. 2024;11:uhae189 39247887 10.1093/hr/uhae189 PMC11377181

[25] Dai Y, Zhang S, Guan J. et al. Single-cell transcriptomic analysis of flowering regulation and vernalization in Chinese cabbage shoot apex. Hortic Res. 2024;11:uhae214 39391013 10.1093/hr/uhae214 PMC11464683

[26] Bai Y, Liu H, Lyu H. et al. Development of a single-cell atlas for woodland strawberry (*Fragaria vesca*) leaves during early Botrytis cinerea infection using single cell RNA-seq. Hortic Res. 2022;9:uhab055 35043166 10.1093/hr/uhab055 PMC8969069

[27] Xin X, Du F, Jiao Y. Plant nuclei isolation for single-nucleus RNA sequencing. Methods Mol Biol. 2023;2686:307–11 37540366 10.1007/978-1-0716-3299-4_15

[28] Osorio D, Cai JJ. Systematic determination of the mitochondrial proportion in human and mice tissues for single-cell RNA-sequencing data quality control. Bioinformatics. 2021;37:963–7 32840568 10.1093/bioinformatics/btaa751 PMC8599307

[29] Du J, Wang Y, Chen W. et al. High-resolution anatomical and spatial transcriptome analyses reveal two types of meristematic cell pools within the secondary vascular tissue of poplar stem. Mol Plant. 2023;16:809–28 36895162 10.1016/j.molp.2023.03.005

[30] Lv K, Liu N, Niu Y. et al. Spatial transcriptome analysis reveals de novo regeneration of poplar roots. Hortic Res. 2024;11:uhae237 39512783 10.1093/hr/uhae237 PMC11540759

[31] Du C, Si Y, Pang N. et al. Prokaryotic expression, purification, physicochemical properties and antifungal activity analysis of phloem protein PP2-A1 from cucumber. Int J Biol Macromol. 2022;194:395–401 34822821 10.1016/j.ijbiomac.2021.11.081

[32] Zheng J, Xi M, Lü Y. et al. Transcriptional analysis provides new insights into cold- and dehydration-tolerance signaling pathways and on regulation of stem cell activity in the vascular cambium of poplar. Plant Mol Biol Report. 2013;31:75–86

[33] Guo Y, Xu H, Wu H. et al. Seasonal changes in cambium activity from active to dormant stage affect the formation of secondary xylem in *Pinus tabulaeformis* Carr. Tree Physiol. 2022;42:585–99 34505153 10.1093/treephys/tpab115

[34] Lauvergeat V, Rech P, Jauneau A. et al. The vascular expression pattern directed by the *Eucalyptus gunnii* cinnamyl alcohol dehydrogenase EgCAD2 promoter is conserved among woody and herbaceous plant species. Plant Mol Biol. 2002;50:497–509 12369625 10.1023/a:1019817913604

[35] Vetal PV, Poirier Y. The *Arabidopsis* PHOSPHATE 1 exporter undergoes constitutive internalization via clathrin-mediated endocytosis. Plant J. 2023;116:1477–91 37638714 10.1111/tpj.16441

[36] Rabeh K, Hnini M, Oubohssaine M. A comprehensive review of transcription factor-mediated regulation of secondary metabolites in plants under environmental stress. Stress Biol. 2025;5:15

[37] Shi J, Gong Y, Shi H. et al. ‘*Candidatus* Liberibacter asiaticus' secretory protein SDE3 inhibits host autophagy to promote Huanglongbing disease in citrus. Autophagy. 2023;19:2558–74 37249424 10.1080/15548627.2023.2213040 PMC10392736

[38] Zhao Y, Yang X, Zhang J. et al. Thaumatin-like protein family genes VfTLP4-3 and VfTLP5 are critical for faba bean's response to drought stress at the seedling stage. Plant Physiol Biochem. 2024;206:108243 38048701 10.1016/j.plaphy.2023.108243

[39] Cawley GF, Connick JP, Eyer MK. et al. Environmentally persistent free radicals stimulate CYP2E1-mediated generation of reactive oxygen species at the expense of substrate metabolism. Drug Metab Dispos. 2025;53:100012 39884817 10.1124/dmd.124.001939

[40] Ho AYY, Day DA, Brown MH. et al. *Arabidopsis* phospholipase Dδ as an initiator of cytoskeleton-mediated signalling to fundamental cellular processes. Funct Plant Biol. 2009;36:190–8 32688638 10.1071/FP08222

[41] Pradhan, Mitra P, Loqué D. Histochemical staining of Arabidopsis thaliana secondary cell wall elements. J Vis Exp. 2014;87:51381 10.3791/51381 PMC4186213 24894795

[42] Milhinhos A, Miguel CM. Hormone interactions in xylem development: a matter of signals. Plant Cell Rep. 2013;32:867–83 23532297 10.1007/s00299-013-1420-7

[43] Xu C, Shen Y, He F. et al. Auxin-mediated Aux/IAA-ARF-HB signaling cascade regulates secondary xylem development in Populus. New Phytol. 2019;222:752–67 30582614 10.1111/nph.15658

[44] Mäkilä R, Wybouw B, Smetana O. et al. Gibberellins promote polar auxin transport to regulate stem cell fate decisions in cambium. Nat Plants. 2023;9:631–44 36997686 10.1038/s41477-023-01360-w PMC10119023

[45] Qian P, Song W, Zaizen-Iida M. et al. A Dof-CLE circuit controls phloem organization. Nat Plants. 2022;8:817–27 35817820 10.1038/s41477-022-01176-0

[46] Liao P, Zeng T, Chen Y. et al. Lemon zinc finger protein ClSUP induces accumulation of reactive oxygen species and inhibits citrus yellow vein-clearing virus infection via interactions with ClDOF3.4. J Exp Bot. 2024;75:7300–16 39185708 10.1093/jxb/erae361

[47] Peng T, Kang J-L, Xiong X-T. et al. Integrated transcriptomics and metabolomics analyses provide insights into the response of Chongyi Wild Mandarin to *Candidatus* Liberibacter Asiaticus infection. Front Plant Sci. 2021;12:748209 34721476 10.3389/fpls.2021.748209 PMC8551615

[48] Moura JCMS, Bonine CAV, de Oliveira Fernandes Viana J. et al. Abiotic and biotic stresses and changes in the lignin content and composition in plants. J Integr Plant Biol. 2010;52:360–76 20377698 10.1111/j.1744-7909.2010.00892.x

[49] Li W, Zuo Y, Deng L. et al. Pine wood Nematode’s migration and defense mechanism of highly resistant and susceptible Pinus massoniana. Forests. 2023;14:2108

[50] Vanholme R, de Meester B, Ralph J. et al. Lignin biosynthesis and its integration into metabolism. Curr Opin Biotechnol. 2019;56:230–9 30913460 10.1016/j.copbio.2019.02.018

[51] Xie M, Zhang J, Tschaplinski TJ. et al. Regulation of lignin biosynthesis and its role in growth-defense tradeoffs. Front Plant Sci. 2018;9:1427 30323825 10.3389/fpls.2018.01427 PMC6172325

[52] Ma W, Pang Z, Huang X. et al. Citrus Huanglongbing is a pathogen-triggered immune disease that can be mitigated with antioxidants and gibberellin. Nat Commun. 2022;13:529 35082290 10.1038/s41467-022-28189-9 PMC8791970

[53] Chaffey N, Cholewa E, Regan S. et al. Secondary xylem development in Arabidopsis: a model for wood formation. Physiol Plant. 2002;114:594–600 11975734 10.1034/j.1399-3054.2002.1140413.x

[54] Baba K, Karlberg A, Schmidt J. et al. Activity-dormancy transition in the cambial meristem involves stage-specific modulation of auxin response in hybrid aspen. Proc Natl Acad Sci USA. 2011;108:3418–23 21289280 10.1073/pnas.1011506108 PMC3044397

[55] Bishopp A, Help H, El-Showk S. et al. A mutually inhibitory interaction between auxin and cytokinin specifies vascular pattern in roots. Curr Biol. 2011;21:917–26 21620702 10.1016/j.cub.2011.04.017

[56] Becht E, McInnes L, Healy J. et al. Dimensionality reduction for visualizing single-cell data using UMAP. Nat Biotechnol. 2018;37:38–44 10.1038/nbt.4314 30531897

[57] Zhou B, Jin W. Visualization of single cell RNA-Seq data using t-SNE in R. Methods Mol Biol. 2020;2117:159–67 31960377 10.1007/978-1-0716-0301-7_8

[58] Butler A, Hoffman P, Smibert P. et al. Integrating single-cell transcriptomic data across different conditions, technologies, and species. Nat Biotechnol. 2018;36:411–20 29608179 10.1038/nbt.4096 PMC6700744

[59] Qiu X, Mao Q, Tang Y. et al. Reversed graph embedding resolves complex single-cell trajectories. Nat Methods. 2017;14:979–82 28825705 10.1038/nmeth.4402 PMC5764547

[60] Xiao Y-X, Dutt M, Ma H. et al. Establishment of an efficient root mediated genetic transformation method for gene function verification in citrus. Sci Hortic. 2023;321:112298

[61] Tian X-B, Luo J, Sun X. et al. Microtubule-mediated defence reaction of grapevine to *Neofusicoccum parvum* via the transcription factor VrWRKY22 promoting the kinesin-like protein VrKIN10C. Int J Biol Macromol. 2025;308:142519 40147667 10.1016/j.ijbiomac.2025.142519

