# Supplementary material for: Single-nucleus transcriptomics reveals the cellular immune responses to Candidatus Liberibacter asiaticus in rough lemon
Source: Hortic Res. 2025 Oct 1;13(1):uhaf265. doi: 10.1093/hr/uhaf265 (PMC12861477; doi:10.1093/hr/uhaf265)
Supplement: Web_Material_uhaf265 [file web_material_uhaf265.zip › Supplymentary Figure 250610.docx]

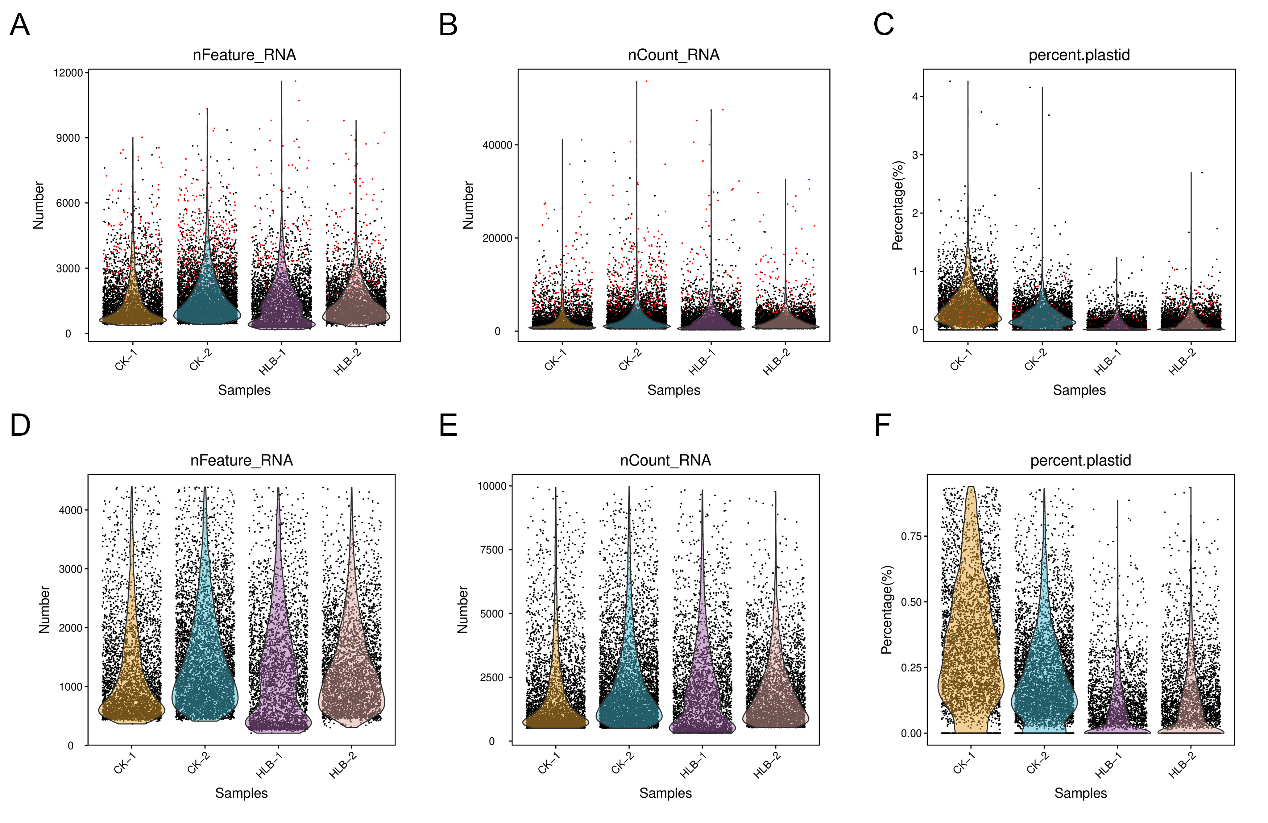


**Supplementary Fig. 1** single-nucleus RNA sequencing data of citrus shoot apex before and after quality control.

**
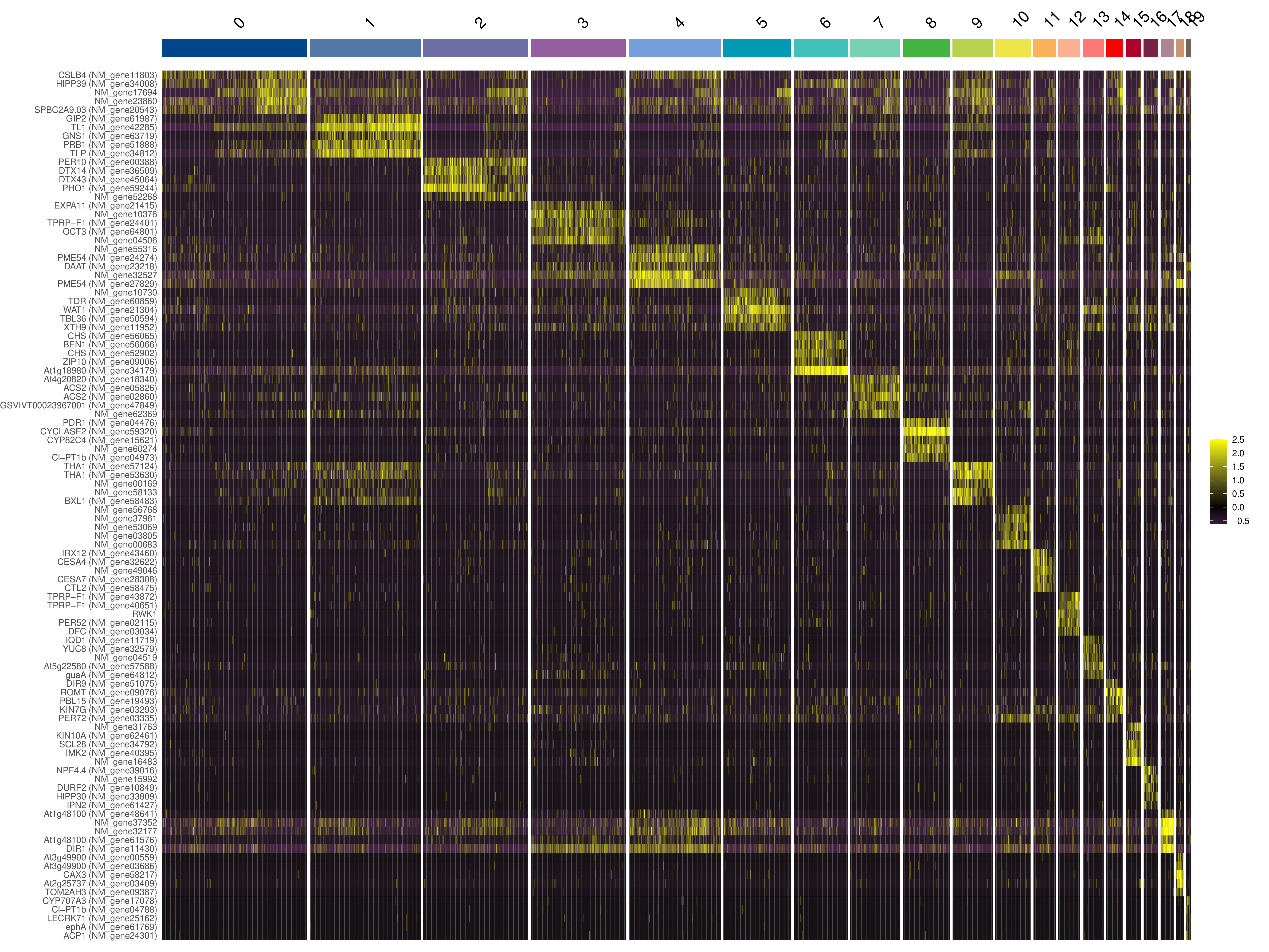
**

**Supplementary Fig. 2** expression patterns of representative genes**.**


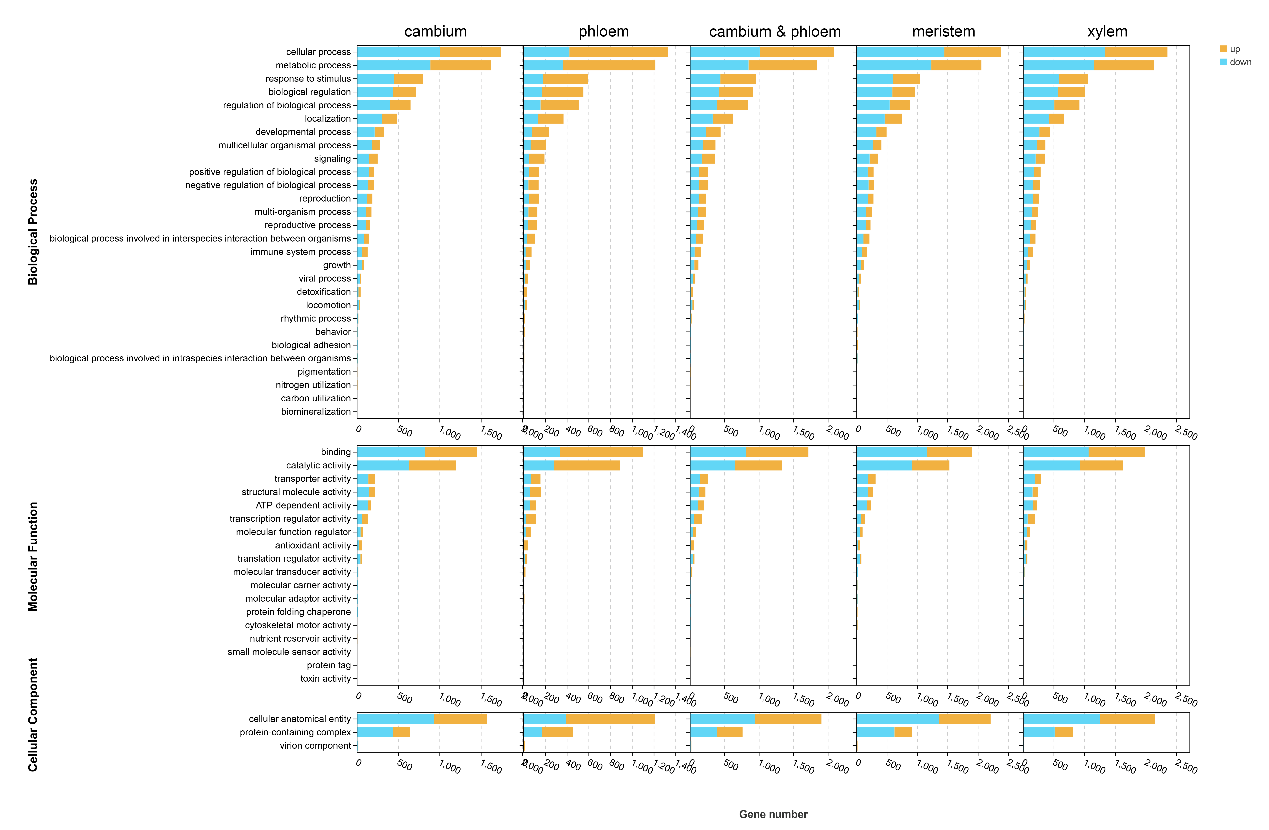


**Supplementary Fig. 3** The number of differentially expressed genes (DEGs) analysis.

**
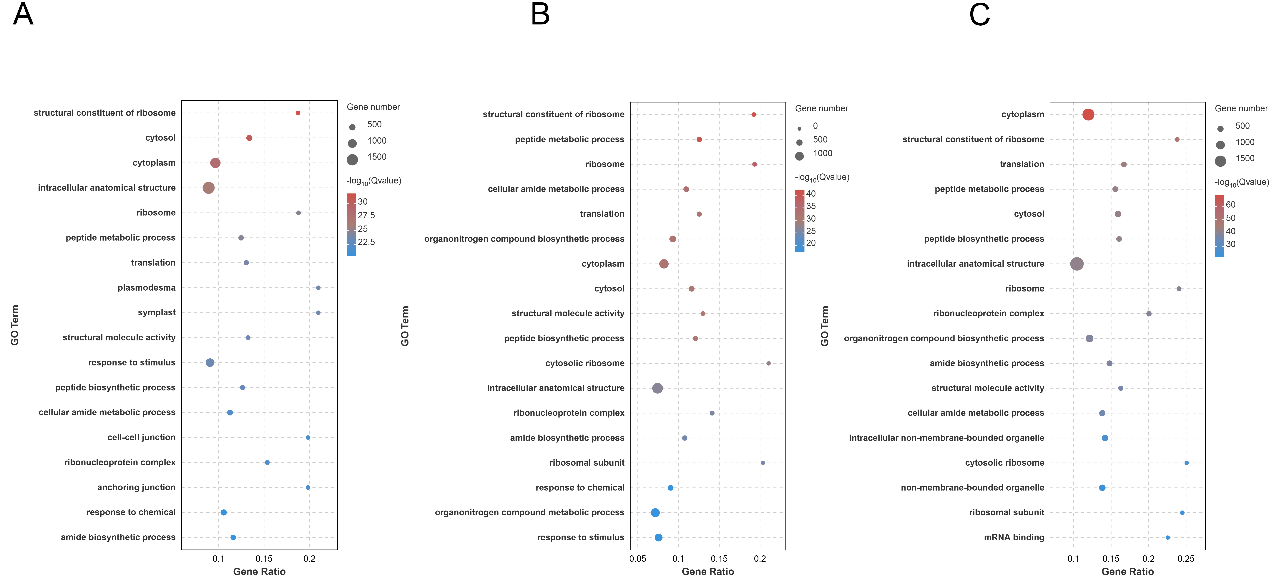
**

**Supplementary Fig. 4** gene Ontology (GO) enrichment of the differentially expressed genes (DEGs) in each cell type.

**
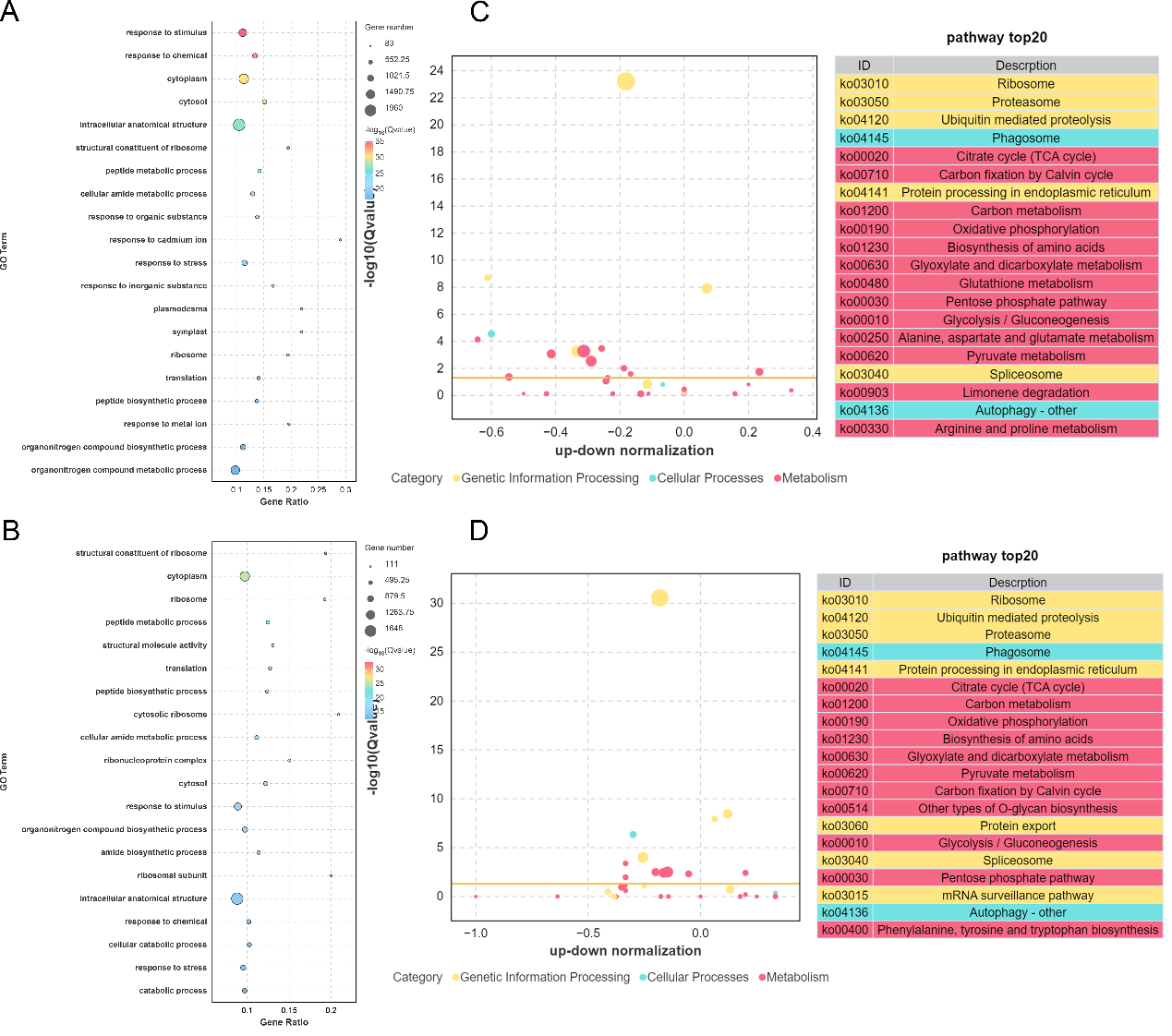
**

**Supplementary Fig. 5** GO and KEGG analysis of cell type 1 and 9

**
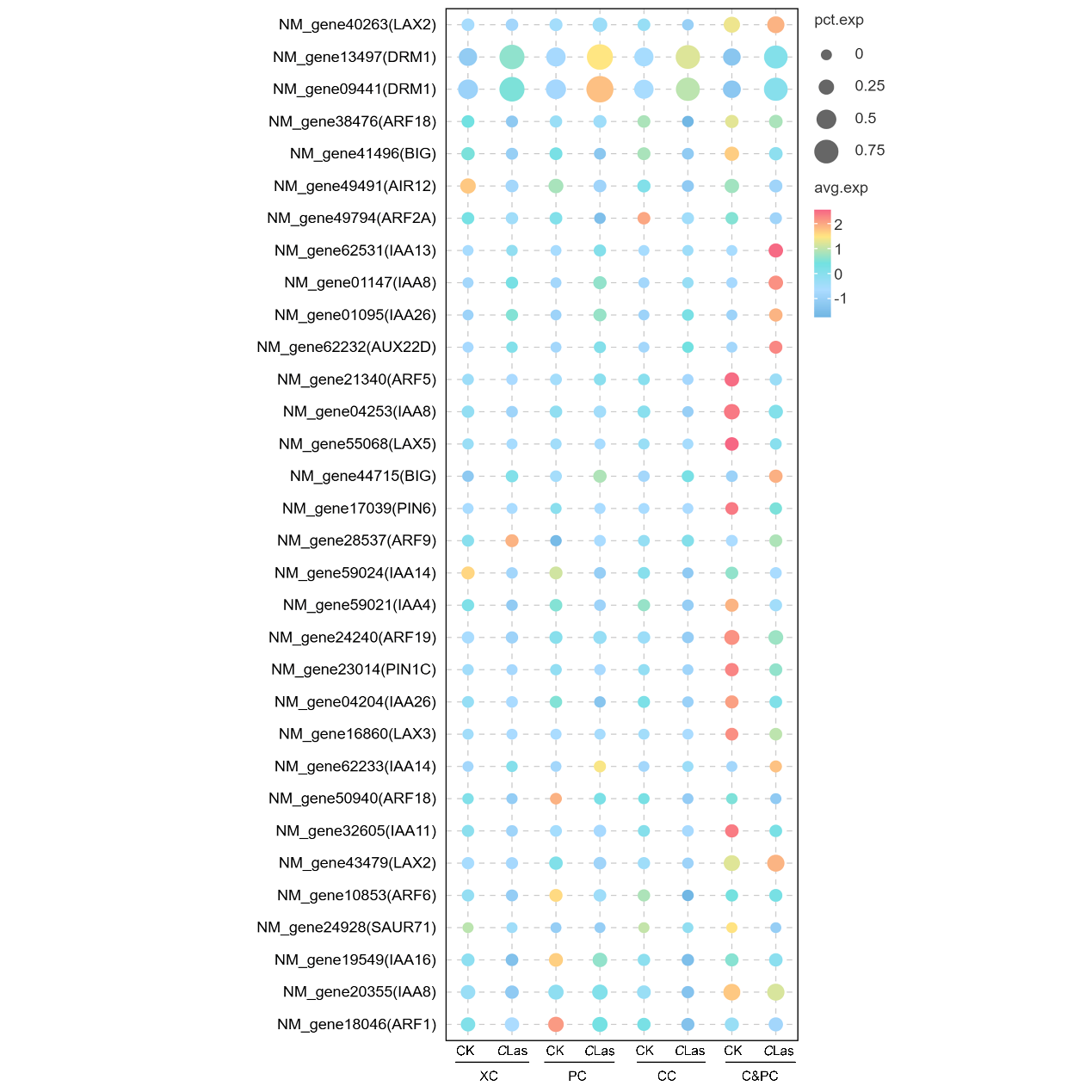
Supplementary Fig. 6** expression patterns of the representative auxin related genes


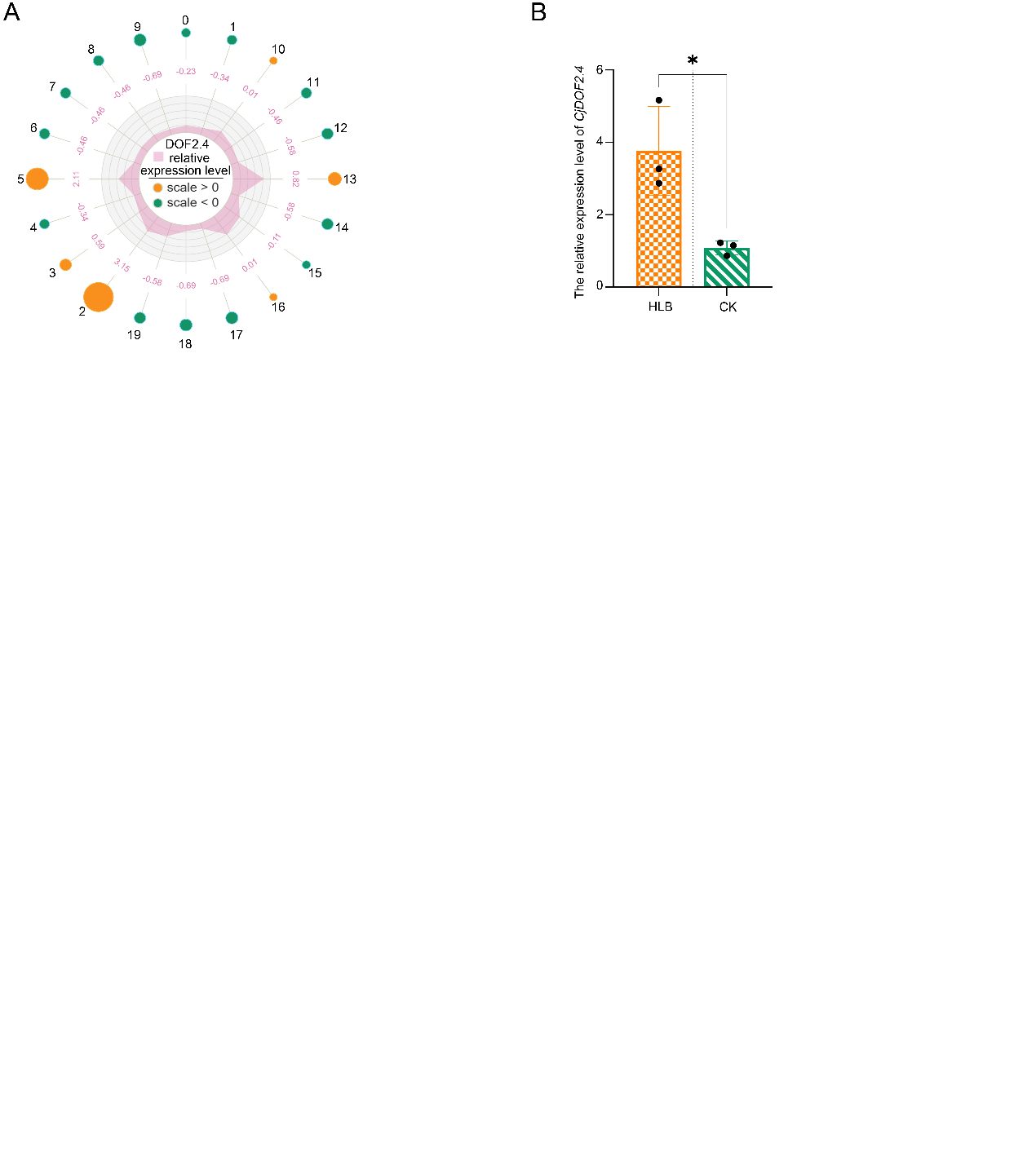


**Supplementary Fig. 7** Analysis of DOF2.4 expression level. (A) The expression of *DOF2.4* in different types of cells in the snRNA-seq. (B) The relative expression level of *DOF2.4* in rough lemon roots infected by *C*Las detected by RT-qPCR.
